# 1167. Hospital Readmissions among Infants Diagnosed with Early-Onset Neonatal Sepsis in Connecticut

**DOI:** 10.1093/ofid/ofab466.1360

**Published:** 2021-12-04

**Authors:** Rebecca Hudon, Vivian Leung, Vivian Leung, Susan Petit, David Banach

**Affiliations:** 1 University of Connecticut School of Medicine, Manchester, Connecticut; 2 Connecticut Department of Public Health, Hartford, CT; 3 UConn Health, Farmington, CT

## Abstract

**Background:**

Early-onset neonatal sepsis, defined as sepsis within 72 hours of birth, results in significant infant morbidity and mortality. Readmissions associated with neonatal sepsis have not previously been well-described. Early-onset neonatal sepsis is a mandatory reportable condition in Connecticut, allowing for expanded data collection through public health surveillance to evaluate readmissions.

**Methods:**

Infants with early-onset neonatal sepsis born in Connecticut during 2007–2016 were identified from statewide surveillance data and matched with a statewide hospital discharge database. We describe readmission rates, causes and timing of readmissions, and demographic and clinical factors associated with readmission among this group.

**Results:**

Among 250 infants with early-onset neonatal sepsis matched to discharge data, 208 (82%) infants survived their initial hospitalization at birth. During the first year of life, 49 (23.6%) infants were readmitted. The most frequent reasons for readmissions were pulmonary complications (19%), systemic symptoms (17%), and gastrointestinal illness (13%). Infants with initial hospitalizations lasting longer than 30 days after birth were associated with higher rates of readmission compared to those discharged within 30 days after birth (35% vs. 19%, p=0.02). Higher readmission rates were observed among non-white infants (29% vs. 18%, p=0.06).

Summary of early-onset neonatal sepsis cases and return hospital visits in Connecticut, 2007-2016

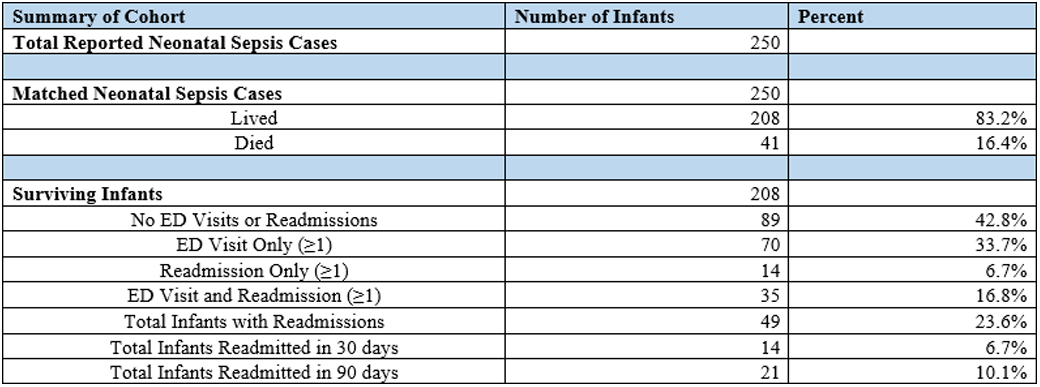

Demographic and clinical factors for Connecticut neonatal sepsis cases, 2007-2016

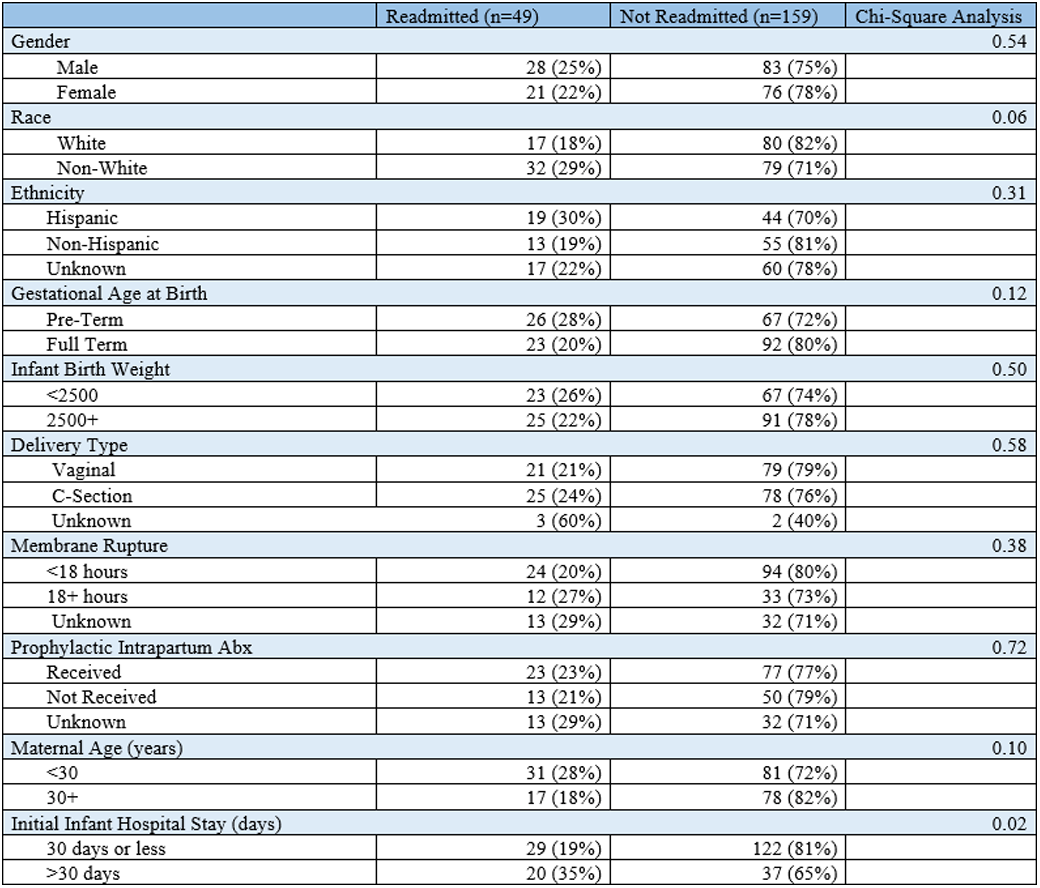

Reason for one-year readmissions of Connecticut neonatal sepsis cases, 2007-2016

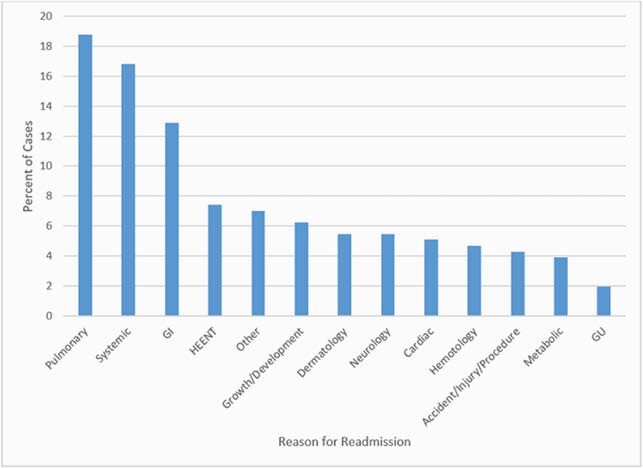

The top three reasons for readmission include pulmonary (19%), systemic (17%), and GI problems (13%).

**Conclusion:**

Given the high proportion of infants diagnosed with early-onset neonatal sepsis who are readmitted within the first year of life, further efforts are needed to prevent readmissions among this vulnerable patient population. Non-white infants and infants with prolonged initial hospitalizations after birth might be at higher risk for readmission. These groups warrant intensified strategies to prevent readmission.

**Disclosures:**

**Vivian Leung, MD**, Nothing to disclose

